# BRIDGE-KO: auditable transfer learning with bridged knockoffs improves cross-cohort microbiome prediction of immunotherapy response

**DOI:** 10.1093/bib/bbag545

**Published:** 2026-10-13

**Authors:** Jie Sun, Zhenjiang Fan, Rebecca Caldino Bohn, Soyeon Kim, Hyun Jung Park

**Affiliations:** Department of Human Genetics, University of Pittsburgh, 4200 Fifth Avenue, Pittsburgh, PA 15260, United States; Department of Adult Neurosurgery, School of Medicine, Stanford University, 450 Jane Stanford Way, Stanford, CA 94305, United States; Department of Human Genetics, University of Pittsburgh, 4200 Fifth Avenue, Pittsburgh, PA 15260, United States; Division of Pediatric Pulmonary Medicine, Children’s Hospital of Pittsburgh, One Children's Hospital Drive, 4401 Penn Ave, Pittsburgh, PA 15224, United States; Department of Human Genetics, University of Pittsburgh, 4200 Fifth Avenue, Pittsburgh, PA 15260, United States

**Keywords:** transfer learning, negative transfer, microbiome data analysis

## Abstract

Transfer learning promises to reuse AI models across biomedical studies, but it often fails when differences between datasets introduce cohort-specific artifacts and ‘negative transfer.’ We present BRIDGE-KO, an interpretable deep-learning framework that turns transfer learning into a testable design process by asking: where to transfer from (source-target coverage and proximity), what to transfer (which representation level is most portable), and how to harmonize data to reduce technical confounding. BRIDGE-KO creates source-preserving knockoffs—synthetic ‘shadow’ features that capture the source dependency structure—and bridges them to a fixed target cohort, providing built-in baselines that flag non-portable signal. Applied to predicting immune checkpoint inhibitor response, transferring gut microbiome profiles from MicroBioMap (n = 168,464) to melanoma immune checkpoint inhibitor cohorts (n = 154), naive baselines performed near chance (AUC ≈ 0.52), whereas batch correction plus proximity-aware transfer improved performance (AUC = 0.67, 10 splits). BRIDGE-KO also enables an interpretable responder-specific audit of microbiome remodeling via correlation-shift spread.

## Introduction

Artificial intelligence is transforming biomedical data science by shifting the focus from building one model per dataset to learning reusable representations that can transfer across studies [1–6]. This shift is enabled by deep neural networks (DNNs) and foundation models that can summarize high-dimensional molecular profiles into compact feature representations, such as cell states [7, 8], regulatory programs [9, 10], or microbial community configurations [11, 12]. These representations can then be transferred to a new target cohort for downstream tasks, such as predicting treatment response or risk scores. This paradigm, known as transfer learning, has broad appeal in computational biology, where labeled outcomes are often limited, expensive, or siloed across cohorts, because only a lightweight task-specific prediction layer must be trained on the target cohort [2, 13]. However, the central practical challenge is avoiding negative transfer-that is, when source-target differences (biological, technical, or demographic) cause the model to rely on non-generalizable patterns (e.g., batch effects or cohort-specific confounding) and perform worse than training on the target cohort alone [14]. In practice, negative transfer is hard to anticipate, quantify, and prevent because the harmful correlations are cohort-specific and are often entangled with true biology. As a result, they are difficult to detect a priori and may only surface when performance is evaluated on the target cohort [14]. Prior work develops transferability metrics, such as LEEP [15], LogME [16], TransRate [17], and OTCE [18] for source/model/layer selection and proposes mitigation strategies-from adversarial filtering of unrelated sources [19], to selective/partial alignment [20] and universal domain adaptation [21]-to reduce negative transfer under domain/task mismatch. Yet, these approaches typically do not identify which specific taxa, pathways, or technical factors drive failure-or how to correct them-in a specific transfer setting. In addition, many conventional transfer-learning strategies assume that the source data can support a supervised pretraining task, which is often not the case for large biomedical reference datasets that lack the target clinical outcome labels.

This limitation is especially consequential in the gut microbiome because it sits at the intersection of high clinical promise and severe, study-specific confounding [22]. On the promise side, the microbiome has been implicated in multiple clinical phenotypes [23], including immune checkpoint inhibitor (ICI) response [24]. Given the clinical impact of ICIs, such as anti-PD-1 therapy, microbiome-based predictors of ICI benefit could provide a non-invasive way to improve patient stratification and treatment selection. At the same time, the microbiome is notoriously difficult to analyze across cohorts: profiles embed many cohort-specific and confounding signals-diet, medications, geography, comorbidities, stool handling, DNA extraction, sequencing, and bioinformatics-so identical taxa- or pathway-level patterns can reflect different mixtures of true biology and confounding. Consistent with this challenge, reported ‘response-associated’ taxa are often inconsistent across studies [25, 26], limiting generalizability and clinical utility. This inconsistency suggests that single-cohort discovery can overfit to cohort-specific signals. Transfer learning offers a potential remedy by leveraging large, general-purpose microbiome resources, such as MicroBioMap [27] and the American Gut Project [28], as source datasets to learn more portable patterns. However, effective use of these resources faces two practical barriers. First, many large-scale microbiome resources are not labeled with the clinical outcomes of interest. General-purpose or healthy-reference microbiome datasets may contain rich information about microbial covariance structure and population variability, but they cannot be used directly for conventional supervised source pretraining, fine-tuning, or adapter-based transfer for ICI response prediction. Second, precisely because confounding is so strong in microbiome data, naive transfer can also amplify non-portable signals and trigger negative transfer. Thus, effective use of large microbiome resources requires methods that can leverage unlabeled source-domain structure while distinguishing transferable biological signal from cohort-specific background structure.

To enable reliable, successful transfer learning for complex biological datasets, we focus on identifying the factors that determine when cross-cohort transfer will generalize-and when it will fail via negative transfer-in gut microbiome prediction (Fig. 1). Although our motivating application is microbiome-based prediction of ICI benefit, the same challenges arise broadly in biomedical settings where cohorts differ in biology, protocols, and context. We therefore aim to enable the design of transfer learning around three practical questions. First, which source samples should be used for transfer? Transfer is more likely to succeed when the source data is sufficiently similar to the target’s biological states and technical setting. This finding motivates mapping target samples into a shared coordinate system (e.g., Geneformer [29] and scGPT [2]). Second, what should be transferred? Transfer performance depends on which model components are reused-ranging from layer-wise fine-tuning [30] and parameter-efficient adapters [31]. Third, how should data be harmonized? Because biomedical cohorts differ in protocols, batch effects, and contextual factors, harmonization is often required so models learn biology rather than artifacts, using approaches from explicit batch correction [32] to latent-space integration (Harmony [33], Scanorama [34]) and generative models that account for technical variation (scVI [7]). Together, these considerations define a design space for transfer learning in biology. Addressing them for a specific source-target pair motivates a deep learning framework that is expressive and scalable, so it can systematically answer these questions.

**Figure 1:**
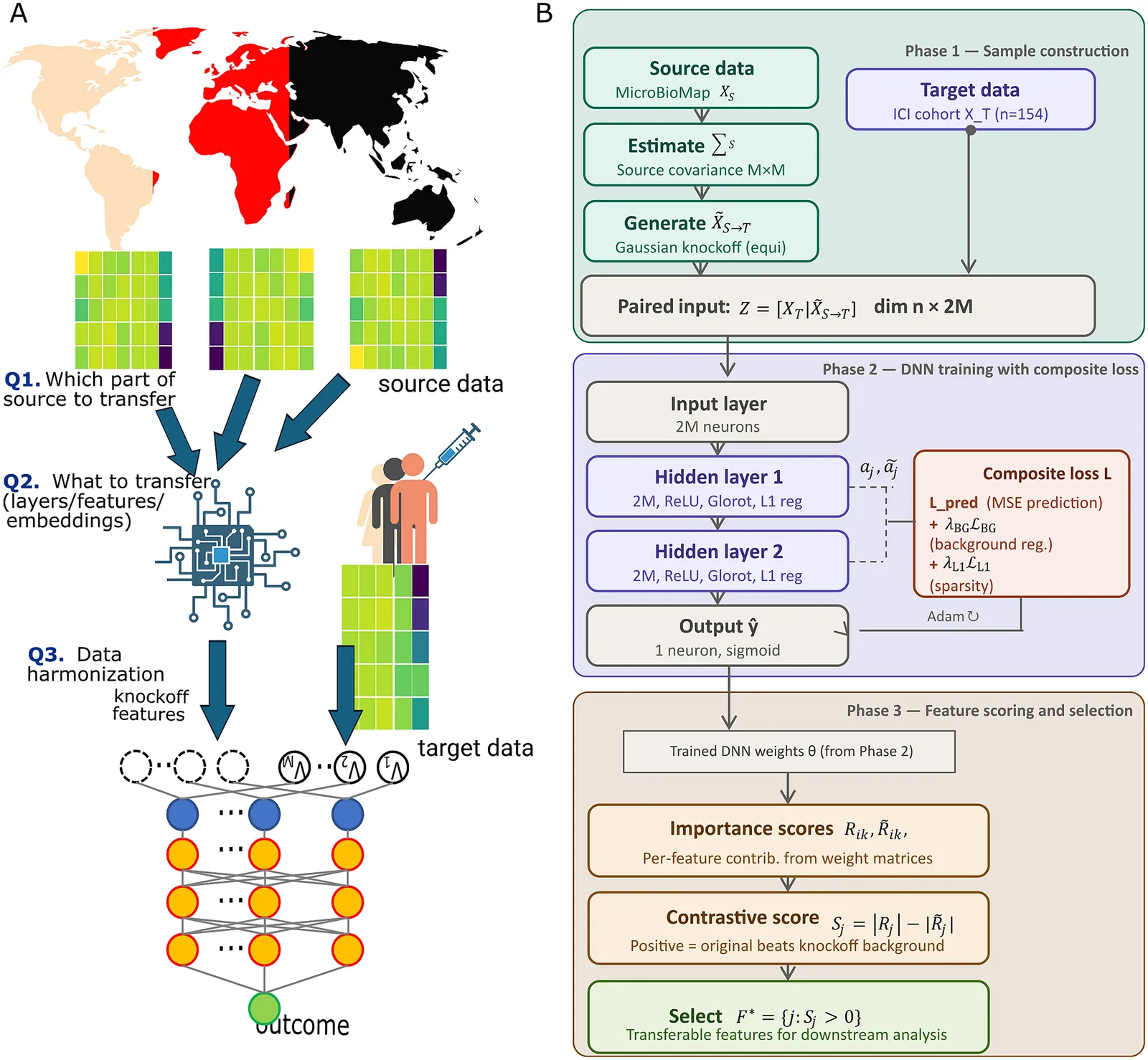
(A) Schematic of the key considerations for effective transfer learning—coverage/proximity (source–target alignment), what to transfer (portable model components), and harmonization (reducing cohort-specific artifacts)—and how BRIDGE-KO operationalizes these choices by learning source dependency structure, generating source-preserving knockoffs, and bridging them to the target cohort to enable scalable, interpretable target prediction and diagnostics for negative transfer.) (B) schematic diagram showing three phases of BRIDGE-KO: The procedure comprises three phases. In Phase 1, the source covariance matrix ${\Sigma}_{\mathrm{S}}$ is estimated from MicroBioMap and used to generate source-informed Gaussian knockoffs for each target sample; the original features and knockoffs are concatenated into a paired input $\mathrm{Z}=[{\mathrm{X}}_{\mathrm{T}} |{\overset{\sim }{\mathrm{X}}}_{\mathrm{S}\to \mathrm{T}}]$ of dimension n × 2 M. In Phase 2, Z is fed into a fully connected DNN with two hidden layers and trained by minimizing the composite loss $\mathcal{L}={\mathcal{L}}_{\mathrm{pred}}+{\lambda}_{\mathrm{BG}}{\mathcal{L}}_{\mathrm{BG}}+{\lambda}_{\mathrm{L}1}{\mathcal{L}}_{\mathrm{L}1}$, which combines supervised prediction with a background-regularization penalty that discourages original features from being indistinguishable from their knockoffs, plus an L1 sparsity term; parameters are updated via Adam. For better interpretation, ${\lambda}_{\mathrm{BG}}$ is currently set to 0 for all reported results, though. In Phase 3, per-feature importance scores derived from the trained weight matrices yield a contrastive score ${S}_{ik}=\mid{R}_{ik}\mid -\mid{\overset{\sim }{R}}_{ik}\mid$ for each taxon, and features with ${S}_j$> 0 are selected as the transferable feature set F^*^.

We present BRIDGE-KO, Background-Regularized Inference for Differential Knockoff Signatures, a scalable and interpretable deep learning framework to enable reliable transfer learning across heterogeneous datasets. In current practice, transfer learning is often applied in an ad hoc manner, lacking a principled way to explore or configure transferability. To provide a principled alternative, BRIDGE-KO generates knockoffs in a source domain and then ‘bridges’ them to the target domain by pairing source-derived knockoffs with target samples. Prior deep learning-knockoff methods typically generate knockoffs within a single dataset to support statistically controlled feature selection-for example, identifying robust biomarkers and disentangling correlated biological signals [35–37]. In contrast, BRIDGE-KO uses source-derived knockoffs as structured background controls to evaluate whether target-predictive signals exceed the covariance-preserving dependency structure learned from a source cohort. A key practical advantage of BRIDGE-KO is that it does not require source-domain outcome labels. Rather than using the source cohort to train an outcome-predictive model, BRIDGE-KO uses unlabeled source data to learn covariance-preserving background structures through knockoffs, which are then bridged to the labeled target cohort. This enables informative transfer from large unlabeled microbiome reference datasets while avoiding the need for source clinical response labels. First (from which part of source to transfer), BRIDGE-KO generates knockoffs in each candidate source and bridges them to the same target cohort, creating a common representation of each source relative to the target. This enables direct comparisons of how source-target similarity-across biological states, feature overlap, and distributional shift-affects transferability and helps identify regimes where transfer breaks down. Second (what to transfer), the bridged knockoff construction yields knockoff-informed signatures as a concrete object to transfer and compare, making it possible to test whether transferring full learned representations, intermediate embeddings, or a compact set of stable predictors yields the most reliable target performance. Third (harmonization), because bridged knockoffs explicitly couple source-derived knockoff structure to target samples, BRIDGE-KO can impose background regularization-a tunable penalty that aligns source and target while suppressing domain-specific artifacts-providing a configurable way to harmonize distributions without assuming a one-to-one mapping of samples or features across cohorts. In this work, we demonstrated BRIDGE-KO to predict ICI outcomes in multiple gut microbiome data collected from melanoma patients undergoing ICI therapies, as a principled, end-to-end design framework that explicitly addresses these three questions. This calibrated perspective is broadly applicable to precision medicine, enabling trustworthy reuse of learned representations for patient stratification, trial enrichment, and mechanism-guided hypothesis generation.

## Results

### BRIDGE-KO, a deep learning framework for lightweight and interpretable transfer learning

BRIDGE-KO begins by training a source-domain encoder to capture feature dependencies-such as microbial co-occurrence or gene expression correlations. Using the learned encoder, it generates knockoff variables, which are synthetic features that preserve the source data’s dependency (correlation) structure. We refer to these source-derived knockoffs as source-KO controls because they preserve source-domain dependency structure while serving as matched background variables for target-domain prediction. These knockoffs serve as feature-wise background controls, matched to each real feature under the source-learned dependency structure. During target-domain training, BRIDGE-KO uses a paired original-knockoff prediction architecture, which we refer to as the contrastive predictor. Here, ‘contrastive’ refers to the structural comparison between each original feature and its matched knockoff control, rather than to a conventional self-supervised contrastive-learning objective. By training on concatenated original and knockoff features, the predictor prioritizes features whose contribution to target prediction exceeds that of their source-KO counterparts. This design helps filter out signals that are indistinguishable from the source-learned background dependency structure.

Because BRIDGE-KO bridges source-preserving knockoffs (source-KO control) to a fixed target cohort, it turns the three transfer-design questions into measurable experiments on the target data performance. (i) Coverage & proximity: BRIDGE-KO can generate the source-KO controls on different parts of the source data as a uniform baseline to quantify when transfer helps versus when transfer improves over target-only training versus when it provides no gain. (ii) What to transfer: BRIDGE-KO can construct source-KO controls at different levels (full representation, intermediate embeddings, or a compact predictor set) to identify the most portable, knockoff-informed signatures. (iii) Harmonization: BRIDGE-KO assesses batch/covariate correction by how strongly it increases the real-versus-KO contrast (e.g., the performance), suggesting better separation of biological signal from cohort-specific artifacts. Importantly, the knockoff-based representation makes these experiments computationally efficient. Instead of retraining large models from scratch for each configuration, BRIDGE-KO reuses the learned source encoder to generate knockoffs that summarize the source background structure. These knockoffs serve as interpretable baselines for a given source-target pairing, allowing the contrastive predictor to be trained quickly on small target datasets. This modular design decouples representation learning from downstream adaptation, enabling fast iteration over transfer settings while preserving knockoff-based control properties. This ensures that limited labels in the target cohort are used efficiently, focusing on true task-specific signals rather than rediscovering generic structure or overfitting cohort-specific noise.

### Correlation-structure concordance defines transferable source subsets for ICI prediction

To evaluate source-target transferability in gut microbiome structure, we ran BRIDGE-KO using a large, population-level reference cohort as the source domain and smaller, clinically rich ICI cohorts as the target. Specifically, we treated MicroBioMap [27] as the source cohort. This source provides broad coverage of gut community structure (168 464 stool samples, 4680 bacterial taxa) and technical variation across various international regions. We then used five U.S. city cohorts of melanoma patients treated with immune-checkpoint inhibitors (ICI; Pittsburgh, New York, Dallas, Houston, Chicago; *n* = 154, see Methods) as the target domain, where labeled outcomes (response (see Methods), survival) are available but sample size is too limited to reliably train a deep model from scratch. This setup reflects a natural transfer-learning scenario: the source provides broad coverage of the taxa and ecological configurations, while the target adds task-specific clinical labels.

Because the target datasets contained 369 taxa at mixed taxonomic ranks, we collapsed both source and target to a shared genus space using the genus intersection across datasets (see Methods), yielding 104 shared genera. We transformed abundance tables into compositional log-ratio space [38] (see Methods). To quantify coverage & proximity between source and target, we summarized each cohort’s dependency structure as a genus-genus correlation matrix and compare source-target concordance. We then estimated Pearson correlations between genus-genus pairs separately in the source and target data to quantify co-variation structure. Most genus pairs showed similar co-variation between source and target (Fig. 2A), but a substantial subset showed clear deviations, consistent with context-specific ecological differences or disease/treatment-associated perturbations [39]. To assess which source subsets (and preprocessing choices) best match the target domain, we re-estimated the source correlation matrix after subsetting MicroBioMap by (i) alternative transformations (e.g., log-transformed abundances; Fig. 2B), (ii) locality (U.S. samples only; Fig. 2C), (iii) sex (Supplementary Fig. 1A, B), and (iv) age groups (Supplementary Fig. 1C, D). The U.S.-only subset improved correspondence with the target, with tighter scatter and higher matrix concordance, as expected because all target samples were collected in U.S. cities and geography is a dominant axis of gut microbiome variation [40, 41]. In contrast, sex- and age-stratified subsets did not enhance alignment (Fig. 2D, Supplementary Fig. 1A–D), consistent with the target’s mixed-sex enrollment and broad age range.

**Figure 2:**
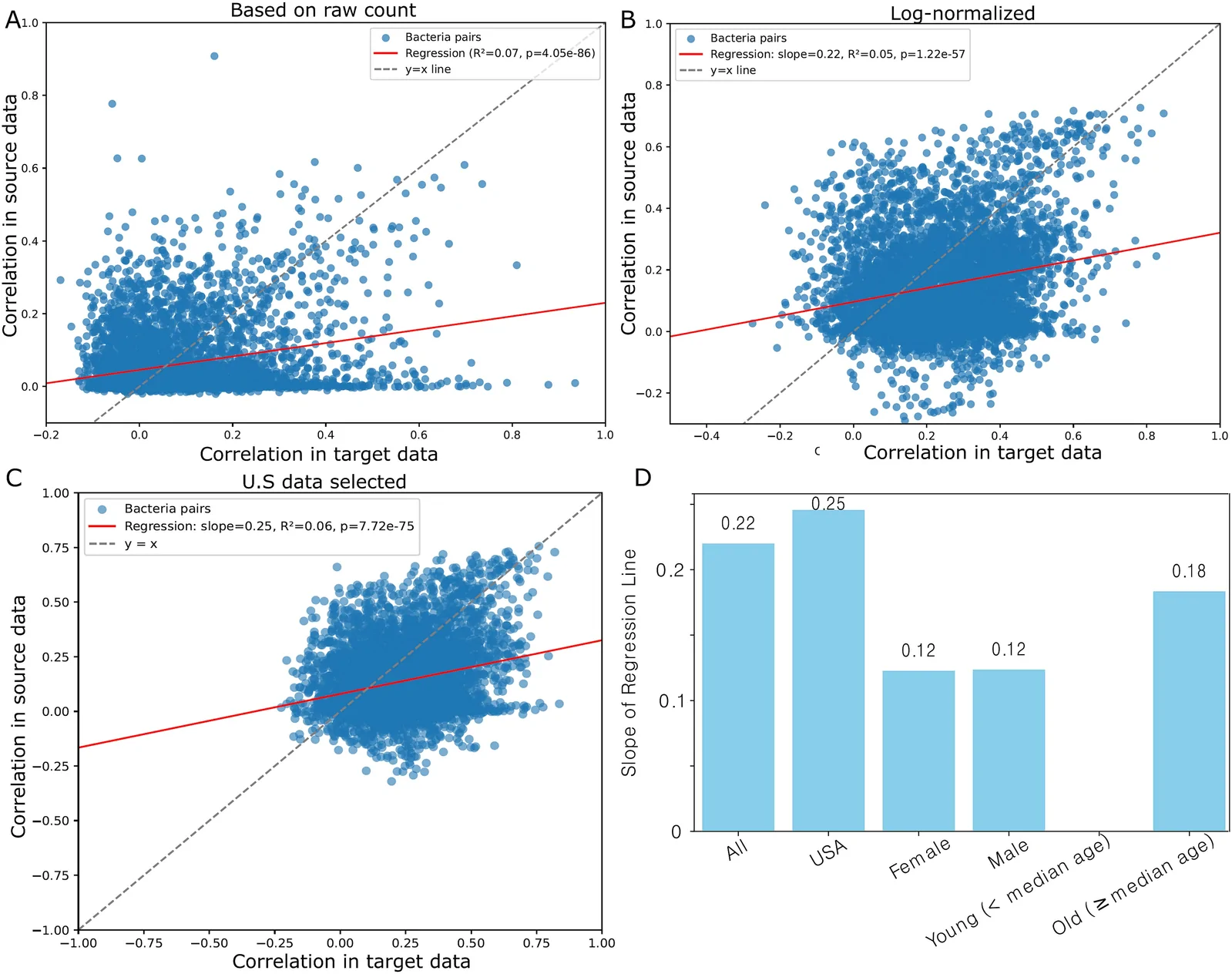
Correlation structure between gut bacteria genus across source and target datasets, when (A) based on the raw counts, (B) after log-normalization, and (C) only on U.S. participants. Dotted lines represent the line of identity (y = x). Red lines represent the linearly regressed line with the R square representing the proportion of the variance explained by the regression line. (D) Barplot of regression slopes across all, United States, female, male, young, old populations.

### Correlation-shift spread reveals responder network reconfiguration and improves source-target contrast

To probe responder-specific microbiome reconfiguration, we focused on correlation shifts involving response-associated (RA) taxa identified previously [42]. Previously, we developed a deep neural network method with a dedicated training module and model-agnostic effect size estimation to identify gut bacteria associated with responses (RA bacteria) from the ICI patient data [42]. For each genus-genus pair, we quantified the shift in Pearson correlation between the source and target datasets. Across all pairs, RA-involving pairs showed larger correlation shifts than non-RA pairs (Fig. 3A-B). In the source data, gut taxa typically exhibit positive correlations, reflecting stable co-occurrence patterns and shared ecological niches [43–45]. In ICI-treated patients, these correlations weaken especially for RA-involving pairs (Fig. 3C-D), indicating a disruption of their interaction network.

**Figure 3:**
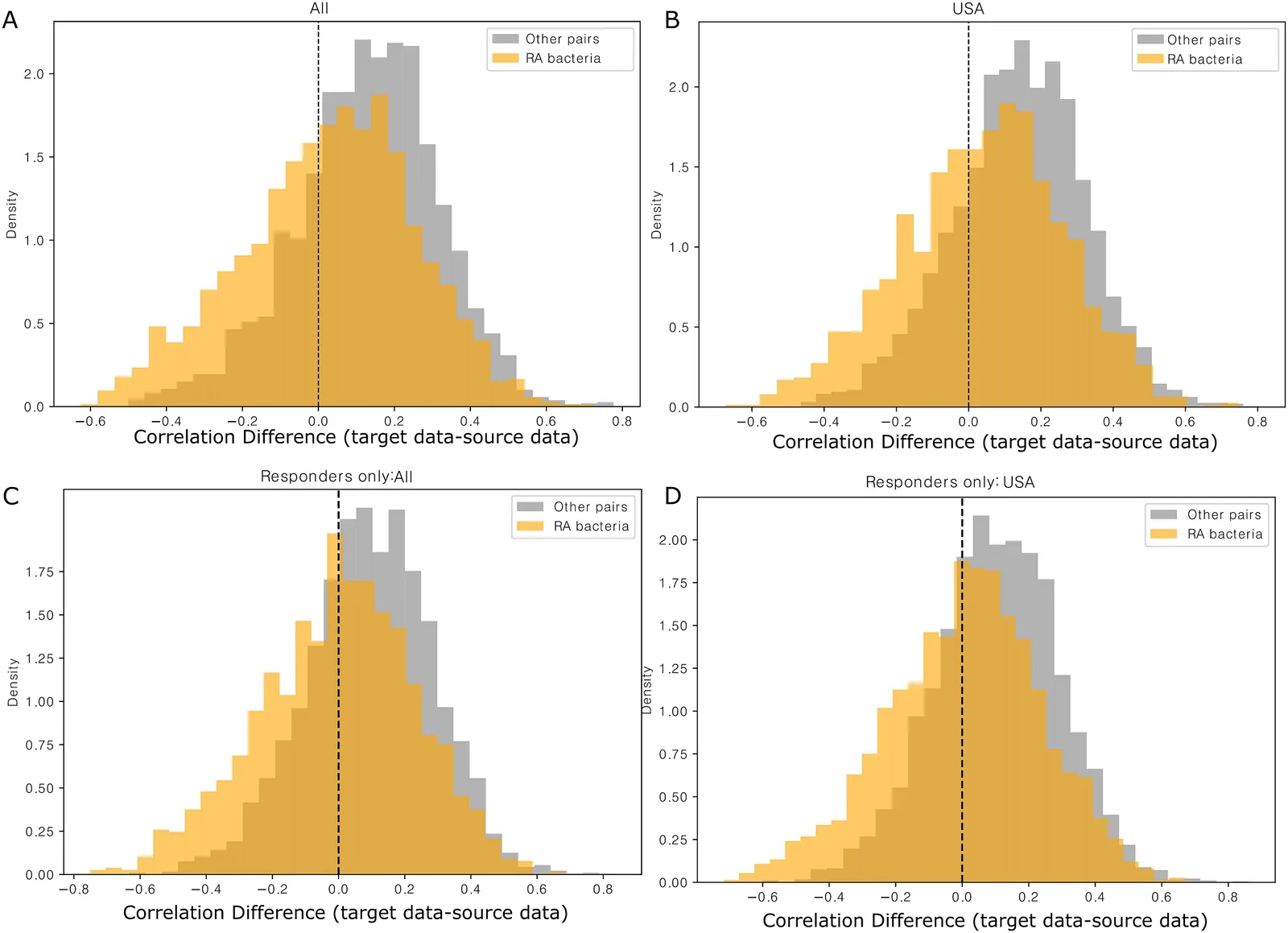
Histogram showing the shift of gut bacteria correlation between source and target data divided by the presence of response-associated bacteria, when (A) all data are used, (B) only U.S. participants are selected, (C) only responders are selected in target data, and (D) only responders are selected in target data and US participants are selected in source data.

To quantify source-target contrast more clearly, we measured the spread of the correlation-shift distribution. In particular, we used distribution width as a simple proxy for source-target discriminability: a wider spread in correlation shifts provides greater contrast, making context-specific co-variation easier to detect. This follows prior work that distributional spread-not only mean shifts-can improve group separability, as in differential correlation and differential variability analyses [46, 47]. Using this measure, RA-involving pairs showed a wider spread of correlation shifts than non-RA pairs (Fig. 3A), consistent with microbiome reconfiguration under ICI treatment.

We next evaluated how source choice influences this RA-specific spread signal. First, the wider spread for RA-involving bacteria pairs persists when the source is restricted by locality (U.S. only; Fig. 3B). As shown in Fig. 2C, this restriction increased source-target correlation concordance, consistent with improved ecological context matching. In contrast, stratifications that reduce source-target concordance (Supplementary Fig. 2A–D) do not broaden the shift distribution and the RA-specific pattern disappears. Second, when we stratified the target cohort by response status, the widened shift distribution was pronounced in responders (Fig. 3C-D) but not in non-responders (Supplementary Fig. 2E–F). This responder-specific pattern supports the interpretation that RA taxa undergo response-associated network reconfiguration under ICI treatment. In sum, we define correlation-shift spread as the width of the distribution of source-target correlation differences across genus pairs. A wider spread indicates greater source-target contrast and may reveal context-specific changes in microbial co-variation. And, the results suggest that correlation-spread is a useful, interpretable metric of source-target contrast that highlights responder-associated microbiome remodeling.

### Proximity-aware source scope and harmonization improve transfer learning for ICI response prediction

To study how source pretraining scope (which portion of the source cohort to pretrain on) influences predictive modeling of immune checkpoint inhibitor (ICI) response, we first evaluated how different knockoff-generation strategies affected target-cohort prediction. First, using Target-KO, in which knockoffs were generated from the target data alone, predictive performance for ICI response across 10 random splits was relatively low (mean AUC ≈ 0.52). This was comparable to using knockoffs generated from the full source cohort, across knockoff generators (Supplementary Fig. 3A–D, **Methods**). These results suggest that neither the broad dependency structure of the full source nor the limited sample size of the target alone is sufficient to enhance predictive accuracy, motivating proximity-aware source selection and harmonization. To design transfer learning around source-target coverage and proximity, we projected source and target samples using genus-level profiles into a shared latent space using UMAP and PCA. Without batch correction, this visualization showed that the ICI target data occupied a distinct region relative to the source samples, indicating substantial source-target distributional shift (Fig. 4A). We ranked source samples by their latent-space distance to the target samples (see Methods), divided them into quartiles (top 25%, 25–50%, 50–75%, and 75–100%; Supplementary Fig. 4A, B), and ran the prediction experiments 10 times. Across the 10 runs, predictive performance did not differ from training with the full source cohort, suggesting that proximity-based subsetting alone is insufficient without harmonization.

**Figure 4:**
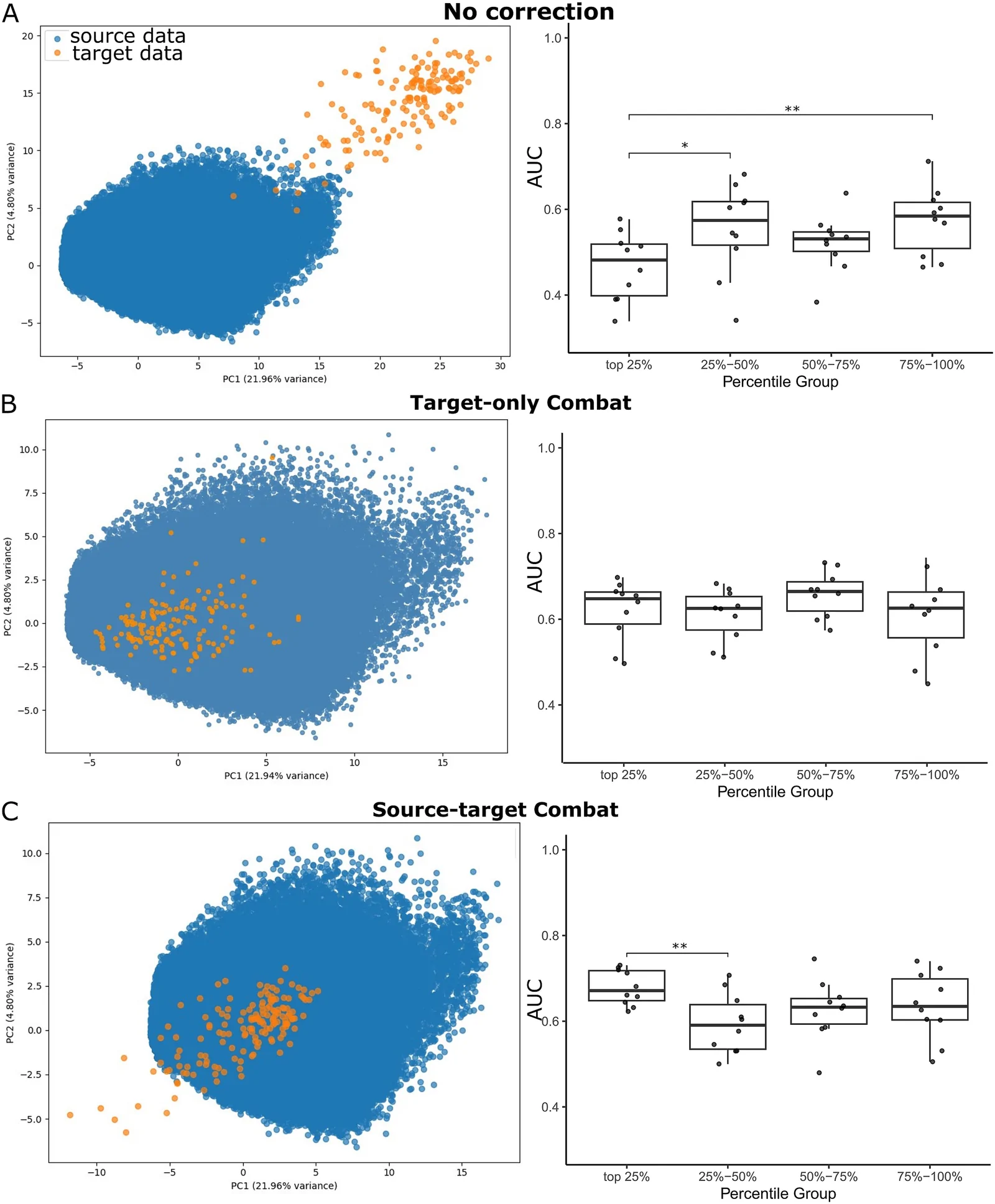
Source–target alignment and transfer-learning performance under different batch-correction settings. (A) without batch correction, (B) after ComBat correction of the target data only, and (C) after ComBat correction of both source and target data. In each component, the left panel displays source–target alignment in PCA space and the right panel shows the corresponding transfer-learning performance. Source samples are shown in blue and target samples in orange. In the boxplots, source samples were ranked by proximity to the target cohort and divided into four percentile groups: Top 25%, 25%–50%, 50%–75%, and 75%–100%. Boxplots show AUC distributions across repeated runs, with individual points representing separate runs, and *P* values indicating pairwise comparisons between source-percentile groups. ^*^ and ^**^ indicates p-value <0.05 and < 0.01, respectively.

To examine the impact of harmonization jointly with source scope, we applied ComBat, which supports explicit covariate modeling, including age and response status (see Methods). First, we applied ComBat only to the target data within each training split (Fig. 4B). This adjustment reduced cohort separation and improved mixing in PCA and UMAP (Supplementary Fig. 4C, D). Performance improved and was stable across proximity ranges. These results suggest that target-only harmonization partially reduces source-target distributional mismatch and stabilizes transfer performance across source-percentile groups. Applying ComBat to both the source and target data further improved source-target alignment in the shared PCA and UMAP spaces and amplified the performance gains (Fig. 4C, Supplementary Fig. 4E, F), especially when training leveraged the most proximal source subset (top 25%). These proximity-based models outperformed models trained on the full source cohort and those using Target-KO alone. Together, these results underscore the value of effective transfer learning and how BRIDGE-KO operationalizes the design of effective transfer learning: by leveraging a proximal subset of a large source cohort with harmonization, we improve prediction in a smaller, domain-specific target cohort.

### BRIDGE-KO improves source-label-free prediction through target-relevant covariance learning

We evaluated BRIDGE-KO’s source-label-free transfer performance through three complementary analyses: comparison with feasible baselines, random-source controls, and source-only versus bidirectional harmonization. First, we compared BRIDGE-KO with baseline approaches feasible in our source-label-free setting, where the large MicroBioMap source cohort provides microbiome profiles but no ICI-response labels (Fig. 5A). The target-only DNN served as a no-transfer reference trained only on the labeled target cohort; CORAL represented covariance-alignment domain adaptation [48]; and DANN represented adversarial domain adaptation that encourages source- and target-invariant representations through domain-discrimination loss [49]. Under the best-performing BRIDGE-KO setting-ComBat applied to both source and target data and using the top 25% most proximal source samples-BRIDGE-KO achieved the highest predictive performance, with a mean AUC of 0.679 (SD = 0.041). This outperformed the target-only DNN without transfer or knockoffs (AUC = 0.625, SD = 0.079), CORAL (AUC = 0.620, SD = 0.046), and DANN (AUC = 0.537, SD = 0.059). The relatively poor performance of DANN suggests that adversarial alignment may be unstable when target supervision is limited. Supervised transferability estimators such as LEEP [15], LogME [16], TransRate [17], and OTCE [18] were not directly applicable because they assume pretrained representations or labeled source tasks, whereas our goal was to select useful samples from an unlabeled source microbiome cohort. Second, we tested whether BRIDGE-KO’s improvement reflected target-informed source selection rather than source sample size alone. In a random-subset control, source subsets were drawn uniformly from the entire MicroBioMap source dataset and size-matched to the PCA-defined proximity groups (top 25%, 25–50%, 50–75%, and 75–100%; Fig. 5B). Source samples in both arms were used only to estimate the feature-feature covariance matrix for knockoff generation and were never added to target training. For the top 25% group, proximity-selected BRIDGE-KO achieved an AUC of 0.679, whereas the size-matched random-source control achieved an AUC of 0.603. Thus, the transfer gain was not explained by source sample size alone, but depended on target-relevant source covariance. Third, we evaluated whether harmonizing only the source data was sufficient. Source-only ComBat produced near-chance AUCs across all proximity groups: 0.501 ± 0.084, 0.475 ± 0.066, 0.513 ± 0.073, and 0.472 ± 0.080 for the top 25%, 25–50%, 50–75%, and 75–100% groups, respectively. In contrast, harmonizing both source and target data yielded the best BRIDGE-KO performance, with an AUC of ~ 0.68. Together, these results show that BRIDGE-KO improves source-label-free prediction by leveraging target-relevant source covariance, and that effective transfer requires both appropriate source selection and bidirectional source-target harmonization.

**Figure 5:**
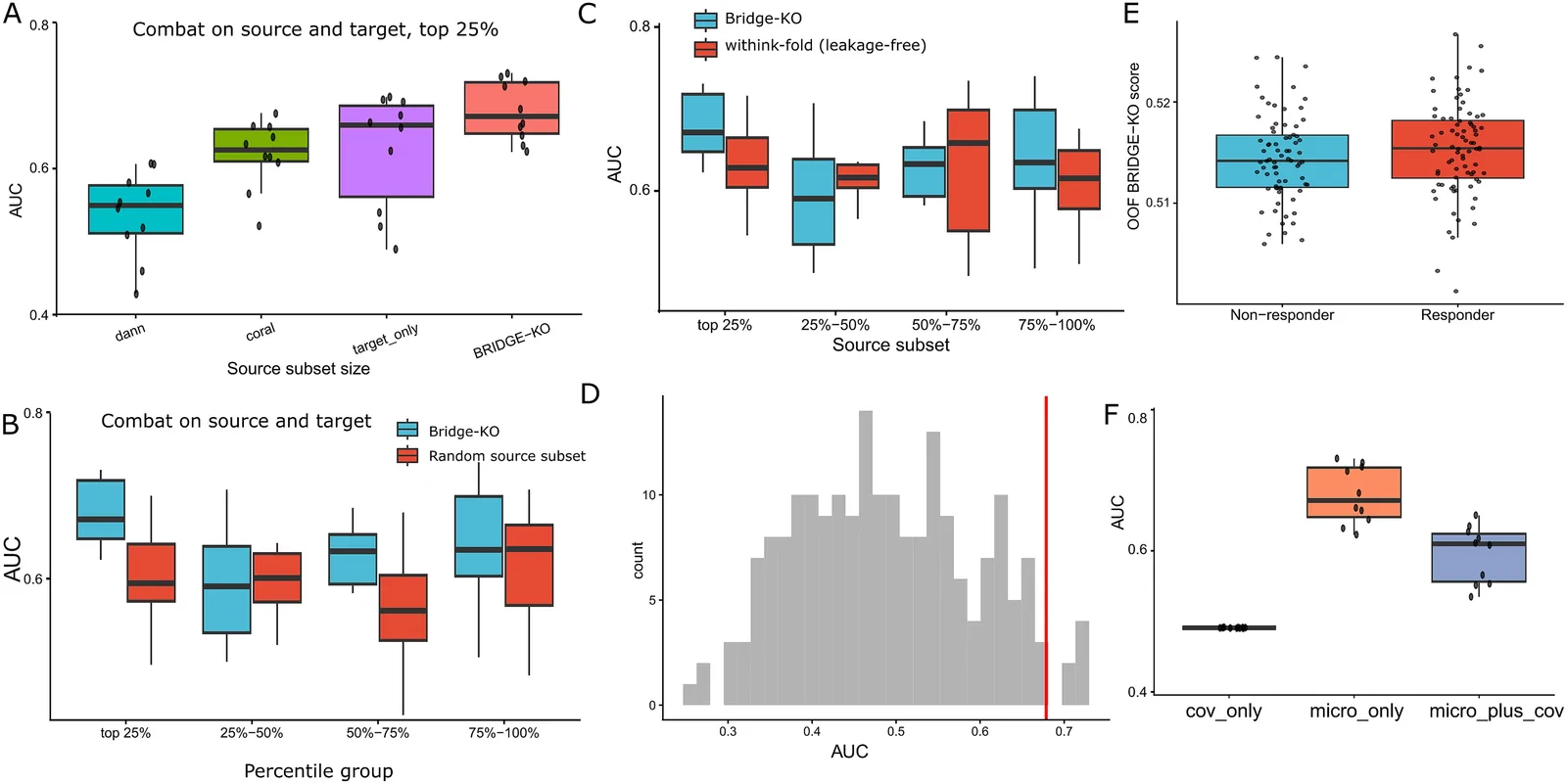
Performance and sensitivity analyses of BRIDGE-KO for source-label-free ICI-response prediction. (A) AUC comparison of BRIDGE-KO with source-label-free baselines: DANN, CORAL, and target-only DNN. BRIDGE-KO used ComBat harmonization of both source and target data and the top 25% most target-proximal source samples. (B) Random-source control comparing BRIDGE-KO with size-matched random source subsets across source-proximity groups. (C) Leakage-free ComBat sensitivity analysis comparing the main BRIDGE-KO setting with within-fold ComBat harmonization. (D) Outcome-label permutation test. The gray histogram shows the permuted AUC null distribution, and the red vertical line indicates the observed BRIDGE-KO AUC. (E) Out-of-fold BRIDGE-KO scores by response group. Each point represents one patient. Responders showed slightly higher scores than non-responders, with substantial overlap. (F) Covariate-aware prediction comparison. Covariates alone performed near chance, while microbiome-only prediction performed better; adding covariates to the microbiome model did not improve AUC.

### Sensitivity analyses show that BRIDGE-KO performance is not explained by leakage, label randomness, or measured covariates

We next performed sensitivity analyses addressing three potential explanations for BRIDGE-KO performance: preprocessing leakage, random-label performance, and confounding by sequencing method or available clinical covariates. First, because the best-performing BRIDGE-KO setting used ComBat harmonization across source and target data, we performed a leakage-free sensitivity analysis in which ComBat was fit within each training fold using only source samples and training-target samples, then applied to held-out target samples (Fig. 5C). Under this stricter evaluation, BRIDGE-KO remained above chance across source groups, with AUCs of 0.623, 0.608, 0.629, and 0.620 for the top 25%, 25–50%, 50–75%, and 75–100% groups, respectively. Although the top-25% AUC decreased from 0.679 to 0.623, the within-fold result indicates that BRIDGE-KO performance was not driven solely by test-sample leakage during harmonization. Second, we tested whether the observed performance exceeded random-label expectation. Across 200 outcome-label permutations of the full pipeline, the permuted null distribution had a mean AUC of 0.489, whereas the observed best-configuration AUC was 0.679, yielding an empirical P-value of 0.040 (Fig. 5D). To quantify uncertainty in the AUC estimate, we also conducted a stratified balanced bootstrap by resampling jointly by city and response status across 10 seeds and 1000 resamples. This analysis yielded a mean AUC of 0.719 with a 95% confidence interval of 0.50–0.88. Thus, although the confidence interval reflects the modest test-set size, the permutation analysis supports that the observed performance is unlikely to arise from random label assignment alone.

Third, we assessed whether available clinical covariates or sequencing batch explained the microbiome signal. Using clinical metadata and cohort sequencing information for our ICI data (Supplementary Table S3), we assembled available age, sex, BMI, sequencing method, antibiotic use, H2RA/PPI use, and pretreatment disease stage metadata for the 154 analysis samples, although availability varied substantially across cohorts (Supplementary Table S6). Antibiotic use and disease stage were collected only in Pittsburgh, and ECOG performance status was unavailable. Supplementary Table S7 reports the exact analysis population, predictors, and missing-data handling for each covariate-only and covariate-adjusted model. Median imputation and ‘unknown’ coding allowed models to retain samples with incomplete covariate data, but did not compensate for variables that were unmeasured in entire cohorts. The five cohorts were profiled using three taxonomic pipelines-JAMS/Last-Known-Taxon for Pittsburgh (n = 63), MetaOMineR for Houston (n = 25), and MetaPhlAn-family pipelines for Chicago, New York, and Dallas (n = 66)-which we encoded as a three-level sequencing-method covariate (Methods). Because each cohort used a single protocol, sequencing method was confounded with site/cohort and corresponded to the batch structure addressed by ComBat. Antibiotic use and stage were available only for Pittsburgh, and ECOG performance status was unavailable. A covariate-only logistic model achieved near-chance prediction (AUC ≈ 0.491), indicating that the available covariates carried little response signal on their own. To test the microbiome score under covariate adjustment without train-on-self contamination, we computed an out-of-fold BRIDGE-KO score for every sample using 5-fold cross-validation repeated across 5 seeds. Responders showed slightly higher out-of-fold scores than non-responders, but with substantial overlap (Fig. 5E), consistent with a modest signal. The BRIDGE-KO score alone showed a positive but non-significant association with response (OR = 1.23 per SD, P-value = 0.22; out-of-fold AUC = 0.57). After adjustment for sequencing method and other available covariates, the effect size was essentially unchanged (adjusted OR = 1.22 per SD, Wald P-value = 0.27; likelihood-ratio test: χ^2^(1) = 1.24, P-value = 0.27), and sequencing method itself was not associated with response. Thus, adjustment for the measured covariates did not materially attenuate the BRIDGE-KO association, although it remained a nonsignificant positive trend. Because several covariates were incompletely observed or available only in Pittsburgh, these analyses cannot exclude residual clinical confounding. We also considered covariate-aware ComBat adjustment. Including response in the ComBat model matrix to preserve responder/non-responder variation was feasible and did not inflate performance. Sequencing method was not added as a separate preservation term because it was identical to the site/cohort batch variable already modeled by ComBat. Other variables, including regimen, antibiotic use, and stage, could not be stably included as preservation terms because their availability was perfectly confounded with the Pittsburgh cohort. Consistent with the covariate-only analysis above, the covariate-only model remained near chance, whereas the microbiome-only model showed higher predictive performance (Fig. 5F). Adding available covariates to the microbiome model did not improve performance or inflate AUC, consistent with the covariate-adjusted regression analysis above. Together, these sensitivity analyses support a conservative interpretation of BRIDGE-KO performance. The fixed 70/30 AUC of 0.679 indicates promising predictive performance, while within-fold harmonization and label permutation argue against preprocessing leakage and random-label artifacts, respectively. Covariate adjustment did not materially attenuate the out-of-fold BRIDGE-KO association, although incomplete and cohort-specific covariate availability prevents exclusion of residual confounding. The fully cross-validated out-of-fold AUC of 0.57 better reflects the modest magnitude of the adjusted predictive signal at the current sample size.

#### Selection-frequency analysis prioritizes stable microbiome candidates

After evaluating prediction performance and sensitivity to leakage, label permutation, and covariate adjustment, we examined whether BRIDGE-KO-selected genera were reproducible across repeated runs. Because BRIDGE-KO contrasts observed microbiome features with source-informed knockoffs, recurrently selected genera may represent stability-supported candidates for downstream interpretation. We therefore summarized feature-selection frequency across 10 BRIDGE-KO runs and treated genera selected in at least six of 10 runs as a descriptive stability tier rather than a formal significance set. To test whether this recurrence exceeded chance expectation, we performed 1000 selection-frequency randomizations that preserved the number of genera selected in each run. The observed number of genera selected in at least six of 10 runs was 42, which fell within the random null distribution (null mean = 38.6; empirical P-value = 0.16). Thus, the ≥6/10 group should be interpreted as a stability-based prioritization tier, not as a globally significant biomarker set. At the individual-genus level, however, *Desulfovibrio* was selected in 10 of 10 runs and exceeded the random expectation under the selection-frequency null (empirical P-value = 0.002). Eight genera were selected in eight of 10 runs (Slackia, Enorma, Gordonibacter, Bacillus, Monoglobus, Mageeibacillus, Turicibacter, and Enterobacter); three of these - Slackia, Enorma, and Gordonibacter - individually exceeded the selection-frequency null (empirical P-value <0.05), while the remainder showed near-threshold recurrence (P-value ≈ 0.05–0.07). We note that these genus-level P-values are not corrected for multiple testing across the 104 candidate genera, so we interpret them as stability-based prioritization rather than formal discoveries. These results support prioritizing highly recurrent genera as candidate microbiome signals for follow-up validation, while avoiding overstatement of feature-selection significance. Together with the model-level analyses, these results support a conservative interpretation of BRIDGE-KO outputs as knockoff-calibrated, stability-supported candidate signals that require validation in larger harmonized cohorts.

### Evidence-based interpretation of selected genera

To validate the biological relevance of features prioritized by BRIDGE-KO, we reran transfer learning under our best-performing configuration: ComBat batch correction applied to both source and target, and pretraining on the most proximal 25% of the source cohort (see Methods). To assess stability under knockoff stochasticity, we repeated the experiment 10 times with different random seeds in BRIDGE-KO. Across runs, BRIDGE-KO selected ~50 genera per run, and all 104 genera were selected at least once across seeds, reflecting seed-to-seed variability in borderline features. We summarized selection stability by frequency across runs, defining frequently selected genera (selected in ≥6/10 runs), intermediate genera (5/10 runs), and infrequently selected genera (≤4/10 runs) (Supplementary Fig. 5; Supplementary Table S2).

We then evaluated whether frequently selected genera have prior evidence for association with immune checkpoint inhibitor (ICI) outcomes. We performed systematic multi-query literature searches followed by manual curation (see Methods), prioritizing evidence from human ICI cohorts/clinical studies and noting mechanistic support when available. Because many studies report species-level associations within a genus, we cite representative species when appropriate. For clarity, we organized the interpretation into three groups: frequently selected taxa with prior response-associated evidence, infrequently selected taxa linked to non-response or weaker evidence, and context-dependent taxa requiring cohort-specific interpretation. Among frequently selected taxa, Akkermansia, Collinsella, and Parabacteroides were repeatedly associated with improved ICI outcomes, whereas Roseburia was more often linked to non-response. For example, *Akkermansia muciniphila* has been enriched in responders across melanoma-inclusive cohorts and supported by mechanistic studies implicating dendritic-cell activation and T-cell priming [26, 50]. *Collinsella aerofaciens* appeared in a responder-associated signature in a seminal melanoma cohort [51], and *Parabacteroides distasonis* was associated with improved outcomes (and, in some reports, reduced toxicity) under combined PD-1 and CTLA-4 blockade [52]. In contrast, *Roseburia intestinalis* was reported at higher abundance in non-responders in early melanoma-focused analyses [53].

Among infrequently selected taxa, Barnesiella, Enterocloster, and Escherichia/Enterobacteriaceae-associated features were more often linked to non-response, while Bifidobacterium was linked to response. *Barnesiella intestinihominis* and Enterobacteriaceae features were incorporated into baseline models predicting non-response in prospective melanoma cohorts [54]. Enterocloster (and related taxa such as Hungatella hathewayi, formerly *Clostridium hathewayi*) were reported as overrepresented among non-responders across multi-cancer cohorts [55, 56]. Conversely, *Bifidobacterium longum* was enriched in responders in Matson et al.’s melanoma cohort and remains a common member of responder-associated consortia [51].

Finally, genera with mixed or context-dependent associations were uncommon among frequently selected taxa (notably Bacteroides) but more prevalent among infrequently selected taxa (e.g., Alistipes, Coprococcus, Lachnoclostridium, Oscillibacter, and Subdoligranulum) [26, 57]. For instance, Bacteroides/Bacteroidales-related taxa were enriched in non-responders in one study [58], whereas other datasets report specific *Bacteroides* species in responders or associated with toxicity under dual-ICI regimens [52]. Together, these results suggest that BRIDGE-KO prioritizes taxa with documented links to ICI outcomes while also surfacing context-dependent genera that may require cohort-specific interpretation.

## Discussion

BRIDGE-KO introduces a principled, end-to-end design framework for transfer learning in biomedicine that makes three recurring decisions explicit and testable: where to transfer from, defined by coverage & proximity, what to transfer, defined by which level of representation yields portable signal, and how to harmonize, defined by how cohort-specific artifacts can be suppressed while preserving biology. In our gut microbiome-ICI case study, BRIDGE-KO uses source-preserving knockoffs bridged to a fixed target cohort to convert these choices into controlled comparisons on held-out target performance. This ‘calibrated’ view of transfer learning is broadly relevant to precision medicine, where models must be reused across cohorts for patient stratification, trial enrichment, and hypothesis generation-without silently importing non-portable patterns.

A key contribution of BRIDGE-KO is that it provides built-in diagnostics for negative transfer through explicit knockoff baselines. When transferred predictors fail to outperform Target-KO (or a broad source-derived knockoff baseline), it flags that the transferred signal is not distinguishable from background structure and supports a conservative ‘do not transfer’ decision. The consolidated ablation and sensitivity analysis further isolated the contribution of each design component (Supplementary Table S5). Target-KO performed near chance (AUC =0.521), while using the full source cohort (0.632) or a size-matched random-source subset (0.603) did not achieve the performance of complete BRIDGE-KO (0.679). Proximity alone without harmonization (0.467) and source-only harmonization (0.501) were ineffective, whereas target-only harmonization provided partial recovery (0.620); the strongest performance required proximal source covariance combined with bidirectional harmonization. No evaluated alternative-including positive ${\lambda}_{BG}$ values, SDP or ASDP knockoff construction, more distant proximity groups, or the CORAL and DANN domain-adaptation baselines-outperformed this complete configuration. Within-fold ComBat remained above chance, and the corresponding covariate analyses showed that clinical variables alone carried little predictive information and did not improve the microbiome model. Together, these comparisons indicate that BRIDGE-KO’s gain was not attributable to source sample size, a particular knockoff algorithm, preprocessing leakage, measured clinical covariates, or generic domain alignment, but to the combined use of target-relevant source covariance and bidirectional harmonization.

The sensitivity analysis **(Supplementary Results,**  Supplementary Table S4) further shows that background regularization controls an operating-point tradeoff rather than universally improving prediction. In the present small-target, high-dimensional setting, increasing.

λ*BG*progressively favored original features over their knockoffs and expanded the selected feature set, but reduced selectivity and did not improve held-out AUC; these findings support λ*BG* =0 for the current application. Positive values may nevertheless merit evaluation when the objective is sensitivity-oriented hypothesis generation rather than parsimonious prediction-for example, when prioritizing microbial taxa or pathways for subsequent experimental validation, retaining multiple members of correlated ecological modules, or screening for weak and distributed community-level signals for which omitting a true contributor may be more costly than carrying additional candidates forward. Such applications would require training-only tuning and independent validation to determine whether the expanded candidate set actually reduces false negatives rather than merely increasing false positives.

Our results further show that coverage/proximity and harmonization interact: proximity-based source subsetting alone did not reliably improve prediction until the source-target mismatch was reduced through harmonization. By projecting source and target samples into a shared latent space and ranking source samples by proximity to the target, we operationalized ‘pretraining scope’ as a tunable design choice. Importantly, ComBat-based harmonization increased source-target mixing in latent space and stabilized performance across proximity ranges; applying ComBat to both source and target amplified these gains, with the strongest performance emerging when transfer leveraged the most proximal source subset. Together, these findings support an actionable principle: portable performance comes from aligning domains and concentrating pretraining on relevant source regions, not from maximizing source size.

Beyond prediction, BRIDGE-KO enables interpretable audits of how microbiome structure differs between source and target. Correlation-structure concordance highlighted geography as a dominant axis of source-target alignment, with a U.S.-restricted source subset better matching U.S. melanoma cohorts than sex- or age-stratified subsets. Building on this, we used correlation-shift spread as a compact, interpretable measure of source-target contrast and responder-specific remodeling: genus pairs involving previously identified response-associated (RA) taxa showed larger correlation shifts and wider shift distributions than non-RA pairs, particularly among responders. This use of distributional spread aligns with prior approaches (e.g., differential correlation and differential variability analyses) that leverage changes in dispersion-not only mean shifts-to reveal context-specific rewiring [46, 47]. While these correlation-based signals are not causal, their responder specificity and dependence on ecological context matching suggest structured, treatment-associated reorganization rather than random variability.

This study has several limitations. First, the target sample size is modest and restricted to melanoma ICI cohorts, limiting power for subgroup analyses (e.g., regimen, antibiotic exposure, site-specific effects) and motivating validation in larger multi-center datasets. Second, collapsing profiles to genus level may obscure species-, strain-, and pathway-level mechanisms; extending BRIDGE-KO to higher-resolution metagenomic features could sharpen biological interpretation and improve portability. Third, our correlation-based analyses provide evidence of structured remodeling but do not establish causality; integrating metabolomics, immune readouts, and prospective interventions will be necessary to connect association to mechanism. Fourth, our source selection relies on unsupervised proximity in latent space; incorporating a priori matching criteria (e.g., diet/medication metadata, protocol harmonization, or leave-one-site-out/federated pretraining) may further reduce negative transfer and improve interpretability.

Although demonstrated here for microbiome transfer to ICI response prediction, the methodological lessons-explicit source-target alignment, harmonization as a first-class design choice, knockoff-calibrated feature selection, and negative-transfer diagnostics via Target-KO baselines-generalize to other biomedical domains such as single-cell, imaging, and multi-omics, where transfer learning is essential for turning scale into reliable clinical utility.

## Methods

### Data harmonization pipeline for transfer learning

We established gut microbiome processing tailored for transfer learning in five steps (Fig. 1). Steps 1–2 perform denoising/quality control, taxonomic and functional profiling, and host-read removal using established tools (DADA2 [59], MetaPhlAn [60], HUMAnN [61]) to ensure community-standard baselines. Step 3 harmonized source and target cohorts into a shared, biologically grounded coordinate system-mapping profiles to a common genus/pathway space with consistent identifiers, units, and metadata-thereby producing transfer-ready matrices so models trained on one cohort could be fine-tuned or evaluated on another without ad hoc remapping. Step 4 applied compositional transformations (CLR and, where appropriate, phylogeny-aware variants) to avoid spurious correlations and place all cohorts in a geometry suitable for machine learning; we additionally performed principled feature refinement (rare-taxa filtering, aggregation) to enhance transferability and interpretability. Step 5 corrected site/platform effects and performed transfer-aware normalization with ComBat/MMUPHin [62, 63], yielding model-ready data in a truly shared coordinate space. Collectively, this pipeline reduced source-target distributional gaps and standardized inputs for downstream transfer learning. Critically, the novelty of our pipeline lies in its transfer-learning native: it turns the key design choices of transfer into explicit, reproducible parameters. First, it specifies which part of the source to use for pretraining by enabling scope-aware subsetting within the harmonized space-either by a priori relevance (e.g., geography or recruitment context) or by latent-space proximity (UMAP/PCA computed after Step 3), so that ‘use all source’ versus ‘use the most relevant source’ is a transparent setting rather than an ad-hoc decision. Second, it defines on which feature space the pretraining subset is chosen: selection can be performed directly on the shared genus/pathway features produced in Step 3 or on outcome-agnostic embeddings derived from those features, avoiding leakage while standardizing how relevance is measured. Third, in Step 5 the pipeline scopes batch correction as an explicit choice-source-only, target-only, or both-with case-aware covariates (e.g., status, age) to preserve biology, allowing users to test whether portability gains arise from stabilizing the source, the target, or symmetric harmonization. Finally, evaluation is built in: beyond predictive metrics (AUC/PR), we include calibration (e.g., Brier score, ECE), robustness audits (repeated splits, external-cohort hold-outs, matrix-concordance of correlation structure, responder-associated pair spread). In sum, rather than stopping at ‘clean data per study,’ the pipeline operationalizes harmonization for transfer by delivering a shared representation, configurable source selection, scoped batch adjustment, and standardized performance auditing-so researchers can systematically tune and justify each transfer decision, summarized in Supplementary Table S1.

### Data collection and normalization

Two datasets were used in the experiments. The first is a large reference dataset from MicroBioMap [27], containing 168,464 samples with 4680 bacterial genera. The second is a small clinical dataset, which is the same dataset used in our paper DeepMicroNET [42], containing 186 samples with 369 bacterial species. Data was collected from five published datasets of different sample size across five locations in the US: Dallas (n = 14), Chicago (n = 39), Houston (n = 25), New York (n = 14), and Pittsburgh (n = 94). To make the dataset comparable in a single study, we normalized and performed the batch-effect-correction on the data sets according to the pipeline developed for this data type [64]. The data was normalized using log2 and quantile transformations. To address batch effects between datasets, we applied the Combat R package (v. 3.20.0) [63] and used UMAP to visualize differences before and after batch correction.

Because the large dataset is annotated at the genus level, we mapped the small dataset to the corresponding genus level. The raw target cohort contained 186 samples. After filtering out unclassified or missing genera, 104 genera were shared between the two datasets. We also filtered out samples in the small dataset with fewer than five non-zero abundance genera, leaving 154 samples. After the filtering, sample size across five locations in the US is Dallas (n = 13), Chicago (n = 39), Houston (n = 25), New York (n = 14), and Pittsburgh (n = 63). Relative abundance was calculated from the raw counts, and log₂-transformation was applied for normalization.

### DNN architecture and knockoff-contrastive prediction

Let X denote the normalized microbiome feature matrix, where n is the number of samples and M is the number of original features: 


$$\mathrm{X}\in \mathbb{R}^{\mathrm{n}\times\mathrm{M}} .$$

For each original feature matrix X, we generated a matched knockoff matrix $\tilde{\mathrm{X}} $, such that each original feature x_j_ had a paired knockoff counterpart $\tilde{\mathrm{x}}_{\mathrm{j}} $. The DNN input was the concatenated original-knockoff matrix, 


$$ [\mathrm{X},\ \tilde{\mathrm{X}}]. $$

so that each sample was represented by both observed microbiome features and their matched background controls.

The DNN consisted of two hidden layers, each matching the input dimension, with ReLU activation, Glorot-normal initialization, and L1 regularization to reduce overfitting. The model was trained using the Adam optimizer with a learning rate of 0.001. The supervised prediction objective was the mean squared error loss, 


$$\mathrm{L}_{\mathrm{pre}}\mathrm{d}=(1/\mathrm{n}){\sum}_{\mathrm{i}=1}^{\mathrm{n}} (\mathrm{y}_{\mathrm{i}}-\hat{\mathrm{y}}_{\mathrm{i}})^2. $$

where y_i_ is the observed outcome and ŷ_i_ is the predicted outcome for sample i.

After training, feature-level association strength was quantified by comparing each original feature with its matched knockoff counterpart. Let R_j_ denote the learned importance score for original feature j, and let $\tilde{\mathrm{R}}_{\mathrm{j}} $ denote the corresponding score for its matched knockoff feature. The knockoff-contrastive effect size was defined as. 


$$ \mathrm{S}_{\mathrm{j}}=|\mathrm{R}_{\mathrm{j}}|-|\tilde{\mathrm{R}}_{\mathrm{j}}|. $$

Positive values indicate that the original feature contributed more strongly to prediction than its matched knockoff control. Features with positive original-versus-knockoff contrastive scores were retained for downstream prioritization. This contrast is knockoff-based and supervised; it is distinct from self-supervised contrastive-learning objectives such as InfoNCE or metric-learning losses.

### Source-informed knockoff construction

For transfer learning, BRIDGE-KO generated knockoff variables using covariance information estimated from the source cohort. For a target cohort X^T^, BRIDGE-KO constructs paired inputs of the form


$$ \left[{X}^T,{\tilde{X}}^{S\to T}\right]\kern-2pt, $$

where ${\tilde{X}}^{S\to T}$denotes knockoff variables generated using the covariance structure learned from the source dataset and applied as a source-informed background control for the target cohort. This design does not require one-to-one matching of source and target samples. Instead, the source dataset contributes a transferable dependency structure, and the target-domain DNN evaluates whether each target feature carries predictive information beyond this source-derived knockoff background.

This source-informed original-knockoff pairing provides the transfer-learning component of BRIDGE-KO. It allows the model to ask whether a target-domain signal is stronger than expected under the dependency structure learned from a larger reference microbiome resource. Therefore, features with positive original-versus-knockoff contrasts are interpreted as candidate transferable signals, whereas features whose contributions are comparable to or weaker than their knockoff counterparts are treated as less likely to represent robust target-predictive information.

To evaluate predictive performance, we randomly split each clinical cohort into a training set comprising 70% of samples and a test set comprising the remaining 30%. Model discrimination was evaluated using the area under the receiver operating characteristic curve (AUC) on the held-out test set. Each scenario was repeated ten times using different random seeds for DNN weight initialization to account for training variability and to ensure robust performance evaluation. A schematic workflow and contrastive predictor diagram were added to illustrate the complete BRIDGE-KO procedure, including covariance estimation, knockoff generation, construction of paired original-knockoff samples, integration into the DNN architecture, supervised training, and derivation of feature-level contrastive scores.

### Gaussian knockoff implementation and method selection

Knockoff variables were generated using the create.gaussian() function from the knockoff R package with a fixed random seed for reproducibility. Because microbiome abundance data are sparse and compositional, the Gaussian knockoff model was treated as an approximation after normalization and scaling. Accordingly, selected genera were interpreted conservatively as knockoff-calibrated, stability-supported candidate signals rather than formal false-discovery-rate-controlled discoveries.

We compared three Gaussian knockoff construction methods implemented in the package: sdp, asdp, and equi. These methods were evaluated in terms of generation time, covariance preservation, original-knockoff self-correlation (Supplementary Fig. 3A, C, D), and downstream AUC (Supplementary Fig. 3B). In the present dataset, equi provided the best overall tradeoff: it was approximately two orders of magnitude faster than sdp and asdp, achieved the lowest mean absolute covariance deviation, and produced the best downstream AUC. Although sdp and asdp yielded lower original-knockoff self-correlation, this did not translate into improved covariance fidelity or predictive performance. Therefore, equi was adopted for all main BRIDGE-KO analyses.

### Source selection, harmonization, and model evaluation

To evaluate target-relevant source selection, source samples were ranked by proximity to the target cohort in PCA space and divided into four percentile groups: top 25%, 25%–50%, 50%–75%, and 75%–100%. For each source group, the selected source samples were used only to estimate the feature-feature covariance matrix used for knockoff generation; source samples were never added to the target training set.

Source and target microbiome profiles were harmonized using ComBat to reduce cohort and sequencing-platform effects. The main BRIDGE-KO configuration used ComBat applied to both source and target data and the top 25% most target-proximal source samples. Additional sensitivity analyses evaluated source-only ComBat, random source subsets, and leakage-free within-fold ComBat.

### BRIDGE-KO framework for source-informed knockoff transfer learning

The DNN architecture in BRIDGE-KO builds on the DeepMicroNET backbone [42], which incorporates model-X knockoff variables as negative controls for identifying robust nonlinear microbiome-phenotype associations. In DeepMicroNET, knockoffs are generated within the analyzed ICI cohort and are used to distinguish response-associated taxa from matched background variables under the dependency structure of that same cohort. Thus, DeepMicroNET is primarily designed for trait-specific feature discovery and network-aware interpretation within a labeled study cohort.

BRIDGE-KO repurposes this DNN/knockoff backbone for a distinct methodological goal: source-label-free transfer learning. Rather than generating knockoffs only within the target ICI cohort, BRIDGE-KO estimates microbiome dependency structure from a large unlabeled source reference dataset and generates source-derived knockoff controls that are paired with target-domain microbiome profiles. In this design, target features are contrasted against a source-informed dependency background, allowing the model to evaluate whether target predictive signals are distinguishable from structured background variation learned from the source cohort. Thus, knockoffs serve not only as negative controls for feature discovery, but also as source-informed background controls for target-domain prediction.

This design introduces transfer-learning components that are not part of DeepMicroNET, including target-relevant source-subset selection, source-target harmonization, and comparison of source-derived knockoff backgrounds across different source regions. These components allow BRIDGE-KO to evaluate which source samples provide useful dependency structure for target prediction and whether harmonization improves the transferability of that structure.

BRIDGE-KO also uses the source-derived knockoff background as a diagnostic for negative transfer. If a transferred predictor does not outperform target-only learning, Target-KO, or full-source-KO controls, the framework supports a conservative interpretation that the transferred signal is not distinguishable from background source-derived dependency structure. This transferability-diagnostic role distinguishes BRIDGE-KO from DeepMicroNET’s primary goal of stable biomarker discovery and network-aware interpretation.

### Background regularization in the contrastive predictor

For transfer learning, BRIDGE-KO uses covariance information estimated from a large source cohort to generate source-informed knockoff controls for target-domain prediction. For a target cohort X^T^, BRIDGE-KO constructs paired inputs of the form $\left[{X}^T,{\tilde{X}}^{S\to T}\right]$ where ${\tilde{X}}^{S\to T}$ denotes knockoff variables generated using covariance structure learned from the source dataset. This design does not require one-to-one matching between source and target samples: the source cohort contributes a transferable dependency structure, while the target-domain DNN evaluates whether each target feature carries predictive information beyond this source-informed knockoff background.

We use the term *contrastive predictor* to refer to this paired original-versus-knockoff prediction architecture and the downstream feature-contrast scoring procedure. Here, ‘contrastive’ refers specifically to the structural comparison between each original feature and its matched knockoff counterpart. It does not denote a conventional self-supervised contrastive-learning objective, such as InfoNCE [65] or metric-learning loss [66]. In BRIDGE-KO, the contrast operates through three components: paired input construction, background-regularized supervised training, and post-training comparison of original and knockoff feature contributions.

**Background regularization** encourages the model to prioritize target-predictive signals whose contribution exceeds the covariance-preserving knockoff background. In the transfer-learning setting, this background can be derived from the source cohort, such as MicroBioMap, and therefore represents dependency structure expected under a larger reference microbiome dataset. The penalty reduces reliance on features whose learned contribution is comparable to or weaker than that of their matched knockoff, because such features may reflect correlated background structure, cohort-specific artifacts, or non-transferable associations.

Let a_j_ and ã_j_ denote learned contribution scores for original feature j and its matched knockoff counterpart, respectively. We define the background-regularization term as ${\mathcal{L}}_{\mathrm{BG}}=\frac{1}{M}\sum_{j=1}^M\max (0,\mid{\tilde{a}}_j\mid -\mid{a}_j\mid +m),$ where m ≥ 0 is a margin parameter. This term penalizes cases in which the knockoff feature has a contribution comparable to or greater than the corresponding original feature, thereby favoring original features that provide predictive information beyond the source- or target-derived knockoff background.

The full training objective is


$$ \mathcal{L}={\mathcal{L}}_{\mathrm{pred}}+{\lambda}_{\mathrm{BG}}{\mathcal{L}}_{\mathrm{BG}}+{\lambda}_{\mathrm{L}1}{\mathcal{L}}_{\mathrm{L}1}, $$

where ${\mathcal{L}}_{\mathrm{pred}}$ is the supervised MSE prediction loss, ${\mathcal{L}}_{\mathrm{BG}}$ is the background-regularization penalty, and ${\mathcal{L}}_{\mathrm{L}1}$ is the L1 weight regularization term. The parameter ${\lambda}_{\mathrm{BG}}$ controls the strength of background regularization, and ${\lambda}_{\mathrm{L}1}$ controls the strength of standard L1 regularization. When ${\lambda}_{\mathrm{BG}}=0$, BRIDGE-KO reduces to the paired original-knockoff DNN without additional background penalization. For interpretation in the results, ${\lambda}_{\mathrm{BG}}$ is currently set to 0 for all reported results. Larger values of ${\lambda}_{\mathrm{BG}}$ impose a more conservative criterion, requiring original features to exceed the knockoff background more strongly before being prioritized.

### Gaussian knockoff implementation and method selection

Knockoff variables were generated using the create.gaussian() function from the knockoff R package with a fixed random seed for reproducibility. Because microbiome abundance data are sparse and compositional, the Gaussian knockoff model was treated as an approximation after normalization and scaling. Accordingly, selected genera were interpreted conservatively as knockoff-calibrated, stability-supported candidate signals rather than formal false-discovery-rate-controlled discoveries.

We compared the three Gaussian knockoff construction methods implemented in the package-sdp, asdp, and equi-with respect to computational cost, covariance preservation, original-knockoff self-correlation, and downstream AUC. In the present dataset, equi provided the best overall tradeoff: it was approximately two orders of magnitude faster than sdp and asdp, achieved the lowest mean absolute covariance deviation, and produced the best downstream AUC (Table 1; Supplementary Fig. 3B, C). Although sdp and asdp yielded lower original-knockoff self-correlation, this did not translate into improved covariance fidelity or predictive performance sufficient to justify the substantially higher computational cost. We therefore adopted equi for all main BRIDGE-KO analyses. Frequently selected genera were largely shared across the three knockoff-generation methods, including 33 genera selected by all three methods, supporting the robustness of the main biological signal to the choice of Gaussian knockoff construction (Supplementary Fig. 3D).

**Table 1:** Comparison of knockoff generation methods on our gut microbiome data (n = 154), train/test protocol (70/30, 10 seeds), and best configuration (PCA-distance source ranking, ComBat applied to both source and target, most-proximal 25% of the source).

Knockoff method	Time (s)	Mean(|cov(X)-cov($\overset{\sim }{X}\Big)$|) (off-diagonal)	Self-correlation	Downstream AUC
equi	9	0.083	0.487	0.679
sdp	1333	0.121	0.172	0.620
asdp	984	0.121	0.172	0.620

#### Batch correction and domain shift analysis

To harmonize the large and small datasets, we applied two types of batch correction: 1) Small dataset only: Correcting for batch effects among the five cohorts contributing to the small dataset. 2) Combined correction: Treating the large and small datasets as two separate batches for correction. Batch correction was performed using ComBat [63]. To assess whether samples in the large dataset that are closer to the small dataset in reduced-dimensional space improve transfer learning performance, we performed PCA and UMAP analyses. The centroid of the small dataset was calculated using Euclidean distance, and the large dataset was divided into four groups based on distance quantiles relative to this centroid.

Although PCoA is commonly used in microbiome analysis [67], it was not applied here due to computational limitations for the large dataset. Knockoff data were then generated for each of the four groups, allowing us to investigate whether distance to the small dataset influences transfer learning performance.

#### Large-language model queries

To investigate the reported functions of each bacterium, we employed a multi-step approach combining automated and manual methods. Specifically, we used large language models (e.g., ChatGPT-5) to generate structured research queries and perform multi-query web searches targeting both the most and least frequently selected bacterial taxa. The outputs were then manually reviewed and curated to ensure accuracy and relevance.

**Key Points**

- Transfer learning in biomedical studies often fails because cohort-specific artifacts can outweigh true biological signal, leading to negative transfer.
- BRIDGE-KO turns transfer learning into a testable framework by evaluating where to transfer from, what representation to transfer, and how to harmonize datasets.
- The method uses source-preserving knockoffs as built-in baselines to detect non-portable signals and reduce misleading transfer.
- In microbiome-based prediction of immune checkpoint inhibitor response, naive transfer performed near chance, whereas BRIDGE-KO with batch correction and proximity-aware transfer improved performance to an AUC of 0.67.
- BRIDGE-KO also provides interpretable insights by auditing responder-specific microbiome remodeling through correlation-shift spread.

## Supplementary Material

Supplementary_materials_bbag545

## Data Availability

https://github.com/sunjie001130/Bridge-KO/
