# Supplementary material for: BRIDGE-KO: auditable transfer learning with bridged knockoffs improves cross-cohort microbiome prediction of immunotherapy response
Source: Brief Bioinform. 2026 Oct 13;27(5):bbag545. doi: 10.1093/bib/bbag545 (PMC13660471; doi:10.1093/bib/bbag545)
Supplement: Supplementary file 1 [file supplementary_materials_bbag545.zip › Supplementary_materials_bbag545_Results.docx]

Supplementary Results

We also examined sensitivity to the background-regularization parameter using the frozen headline configuration and the same ten paired data splits and initialization seeds. The $\lambda_{BG}=0$ condition reproduced the headline results run-for-run. Increasing $\lambda_{BG}$ produced the intended mechanistic effect: mean $L_{BG}$ decreased monotonically, while the fraction of genera for which the original-feature contribution exceeded its knockoff contribution increased from 0.498 at $\lambda_{BG}=0$ to 0.979 at $\lambda_{BG}=1$ (Supplementary Table 4). However, this increasingly favored original features broadly rather than selectively. The mean number of selected genera increased from 51.8 to 101.8 of 104, and overlap with the $\lambda_{BG}=0$ selection decreased accordingly. Consistent with this loss of selectivity, no non-zero value improved held-out performance: mean AUCs were 0.614, 0.596, 0.647, and 0.634 for $\lambda_{BG}=$ 0.001, 0.01, 0.1, and 1, respectively, compared with 0.679 at $\lambda_{BG}$=0 (Supplementary Table 4). Thus, background regularization operated as designed but provided no predictive benefit in this small-target, high-dimensional setting. We therefore retained it as a tunable component of the framework while using $\lambda_{BG}$=0 for the reported application.
