# Supplementary material for: BRIDGE-KO: auditable transfer learning with bridged knockoffs improves cross-cohort microbiome prediction of immunotherapy response
Source: Brief Bioinform. 2026 Oct 13;27(5):bbag545. doi: 10.1093/bib/bbag545 (PMC13660471; doi:10.1093/bib/bbag545)
Supplement: Supplementary file 1 [file supplementary_materials_bbag545.zip › Supplementary_materials_bbag545_Methods.docx]

# Supplementary Methods

## Supplementary Methods 1. Model-X knockoff construction

Let X denote the normalized microbiome feature matrix:

X ∈ ℝⁿˣᴹ

where n is the number of samples and M is the number of original features. For each original feature matrix X, a matched knockoff matrix X̃ was generated so that each original feature xⱼ had a paired knockoff counterpart x̃ⱼ. Knockoff variables serve as matched background controls that preserve the dependence structure of the original features while remaining conditionally independent of the outcome given the original data.

The defining exchangeability condition of model-X knockoffs is that, for any subset of features A ⊆ {1, ..., M}, swapping the original and knockoff variables in A leaves the joint distribution unchanged:

[X, X̃]swap(A) ≡d [X, X̃]

where ≡d denotes equality in distribution. Knockoff variables are also constructed to satisfy

X̃ ⟂ Y | X

where Y is the outcome. These properties justify using knockoff variables as feature-wise negative controls.

For Gaussian model-X knockoff construction, the normalized feature matrix was approximated as following a multivariate Gaussian distribution,

X ∼ N(0, Σ)

where Σ is the covariance matrix of the normalized features:

Σ ∈ ℝᴹˣᴹ

Under this approximation, a valid Gaussian knockoff construction has joint covariance

Cov([X, X̃]) = [[Σ, Σ − D], [Σ − D, Σ]]

where D = diag(s), and s is a nonnegative vector chosen so that the joint covariance matrix is positive semidefinite. Equivalently, the conditional distribution of the knockoff variables given the original variables is

X̃ | X ∼ N(X − XΣ⁻¹D, 2D − DΣ⁻¹D)

Although normalization and scaling place genus-level abundance profiles closer to the Gaussian setting, microbiome data remain sparse and compositional. We therefore interpret selected genera as knockoff-calibrated, stability-supported candidate signals rather than as discoveries with formal model-X false-discovery-rate guarantees.

## Supplementary Methods 2. DNN architecture and feature scoring

The BRIDGE-KO DNN input layer contained 2M neurons, corresponding to the concatenated original-knockoff input [X, X̃]. Each training sample was therefore represented as a paired original-knockoff vector. The model used two hidden layers, each matching the input dimension, with ReLU activation, Glorot-normal initialization, and L1 regularization. Training used the Adam optimizer with a learning rate of 0.001 and the mean squared error prediction loss,

Lₚᵣₑd = (1/n) ∑ᵢ₌₁ⁿ (yᵢ − ŷᵢ)²

After training, BRIDGE-KO computed original-versus-knockoff feature scores. Let Rⱼ denote the learned importance score for original feature j, and R̃ⱼ denote the score for its knockoff counterpart. The knockoff-contrastive score was

Sⱼ = |Rⱼ| − |R̃ⱼ|

Positive Sⱼ values indicate that the original feature contributed more strongly to prediction than its matched knockoff control. Feature importance scores were computed by pairing each original input variable with its corresponding knockoff variable and using matrix operations across the trained DNN layers to quantify relative contribution. Features with positive original-versus-knockoff scores were retained for downstream prioritization.

## Supplementary Methods 3. Source-informed knockoff transfer

BRIDGE-KO differs from within-cohort knockoff analysis by estimating the feature dependency structure from a large unlabeled source cohort and using that source-derived structure to generate knockoff controls for target-domain prediction. For a target cohort Xᵀ, BRIDGE-KO constructs

[Xᵀ, X̃ˢ→ᵀ]

where X̃ˢ→ᵀ denotes source-informed knockoffs generated using covariance information learned from the source dataset. The source data are not added to the target training set. Instead, they contribute only the covariance structure used to generate matched knockoff controls for target samples.

This source-informed pairing enables BRIDGE-KO to ask whether target-domain predictive signals are stronger than expected under the dependency structure learned from a larger reference microbiome resource. Features with positive original-versus-knockoff contrasts are interpreted as candidate transferable signals, whereas features whose contributions are comparable to or weaker than their knockoff counterparts are treated as less likely to represent robust target-predictive information.

## Supplementary Methods 4. Comparison of Gaussian knockoff-generation methods

Knockoff variables were generated using the create.gaussian() function from the knockoff R package. A fixed random seed was used to ensure reproducibility. To enable direct comparison across covariance specifications, we modified the sigma parameter in create.gaussian() to accept different covariance matrices.

We evaluated three Gaussian knockoff construction methods: sdp, asdp, and equi. The sdp method optimizes the knockoff construction but is computationally intensive. The asdp method provides a scalable approximation to sdp, reducing runtime and memory use while retaining similar covariance fidelity. The equi method uses an equicorrelated construction and is computationally efficient.

Each method was evaluated using generation time, mean absolute covariance deviation between original and knockoff covariance matrices, original-knockoff self-correlation, and downstream AUC. In the present dataset, equi provided the best overall tradeoff. It was approximately two orders of magnitude faster than sdp and asdp, achieved the lowest mean absolute covariance deviation, and produced the best downstream AUC. Although sdp and asdp produced lower original-knockoff self-correlation, this did not improve downstream performance. We therefore adopted equi for all main analyses.

To assess robustness of biological interpretation, we compared frequently selected genera across the three knockoff methods. The methods recovered largely overlapping selected genera, including 33 genera shared by all three methods, supporting the stability of the main candidate signals to the choice of Gaussian knockoff construction.

## Supplementary Methods 5. Source-subset selection and random-source control

Source samples were ranked by proximity to the target cohort in PCA space and divided into four percentile groups: top 25%, 25-50%, 50-75%, and 75-100%. For each group, source samples were used only to estimate the covariance matrix for knockoff generation. Source samples were never used as labeled training samples.

To determine whether performance gains reflected source proximity rather than source sample size, we performed a random-source control analysis. For each PCA-defined source group, a random subset of the same size was drawn uniformly from the entire source dataset. Random subsets were drawn from the full source pool rather than from within each PCA band so that the control removed target-proximity selection while preserving sample count.

## Supplementary Methods 6. Source-target harmonization and leakage-free ComBat analysis

Source and target microbiome profiles were harmonized using ComBat to reduce cohort and sequencing-platform effects. The main analysis applied ComBat to the combined source-target matrix before source covariance estimation and BRIDGE-KO modeling.

Because global harmonization can allow held-out target samples to influence the estimated batch-correction parameters, we performed a leakage-free sensitivity analysis. In this analysis, ComBat was fit within each training fold using only source samples and training-target samples. The learned adjustment was then applied to the held-out target test samples. This prevented held-out target samples from contributing to the harmonization model while preserving the downstream BRIDGE-KO workflow.

We also evaluated source-only ComBat, in which only the source data were adjusted toward the target cohort before covariance estimation. This analysis tested whether one-sided harmonization was sufficient to recover transferable covariance structure.

## Supplementary Methods 7. Baseline transfer-learning comparisons

BRIDGE-KO was compared with baseline methods feasible in the source-label-free setting. The target-only DNN was trained only on labeled target samples and used no source information or knockoff variables. CORAL was included as a covariance-alignment domain-adaptation method that aligns second-order feature statistics between source and target data without requiring source outcome labels. DANN was included as an adversarial domain-adaptation baseline that encourages domain-invariant representations through a domain-discrimination loss. Because the source cohort lacked ICI-response labels, the DANN-style baseline used source and target samples for domain discrimination and labeled target samples for response prediction.

Supervised transferability estimators such as LEEP, LogME, TransRate, and OTCE were not applied because they assume pretrained representations or labeled source tasks, whereas the BRIDGE-KO setting involves an unlabeled source microbiome cohort and a smaller labeled target cohort.

## Supplementary Methods 8. Outcome-label permutation and bootstrap uncertainty analysis

To test whether predictive performance exceeded random-label expectation, we performed an outcome-label permutation analysis. Target response labels were randomly permuted, and the full BRIDGE-KO pipeline was repeated for each permutation. The observed best-configuration AUC was compared with the permuted null distribution to compute an empirical p value.

To quantify uncertainty in the AUC estimate, we conducted a stratified balanced bootstrap of the test AUC. Resampling was performed jointly by city and responder/non-responder status across 10 random seeds and 1,000 bootstrap resamples. This design preserves both site structure and response balance during resampling and provides an uncertainty estimate that reflects the modest test-set size.

## Supplementary Methods 9. Covariate adjustment and out-of-fold BRIDGE-KO score

To assess whether the BRIDGE-KO microbiome signal was explained by measured clinical or technical covariates, we joined available covariates to all 154 target samples. Covariates included age, sex, BMI, sequencing method, antibiotic use, H2RA/PPI use, and pre-treatment disease stage. Sequencing method was encoded as a three-level covariate corresponding to the taxonomic profiling pipelines used by the cohorts. Because each cohort used a single profiling protocol, sequencing method was confounded with site/cohort and corresponded to the batch structure addressed by ComBat. Antibiotic use and disease stage were available only for the Pittsburgh cohort, and ECOG performance status was unavailable.

We first fit a covariate-only logistic regression model to evaluate whether measured covariates predicted response. We then computed an out-of-fold BRIDGE-KO score for every sample using 5-fold cross-validation repeated across 5 random seeds. Each sample received a score only from models in which that sample was held out, preventing train-on-self contamination.

To evaluate whether the microbiome score contributed information beyond measured covariates, we fit nested logistic regression models:

M₀: response ∼ covariates

M₁: response ∼ covariates + BRIDGE-KO score

The BRIDGE-KO score was standardized before modeling, and the effect was reported as an odds ratio per standard deviation increase. Model comparison was performed using a likelihood-ratio test, and the BRIDGE-KO coefficient was evaluated using a Wald test.

## Supplementary Methods 10. Covariate-aware ComBat analysis

We also considered covariate-aware ComBat adjustment. Including response status in the ComBat model matrix to preserve responder/non-responder variation was feasible and did not inflate performance. Sequencing method was not included as an additional preservation term because it was identical to the cohort/site batch variable already modeled by ComBat. Other variables, including regimen, antibiotic use, and disease stage, could not be stably included as preservation terms because they were populated only for the Pittsburgh cohort, making their availability perfectly confounded with site. We therefore treated this as a data limitation rather than forcing an unstable covariate-aware harmonization model.

## Supplementary Methods 11. Selection-frequency randomization

To evaluate whether recurrently selected genera exceeded chance expectation, we performed a selection-frequency randomization analysis. Across 1,000 randomizations, we preserved the number of genera selected in each of the 10 BRIDGE-KO runs and randomly reassigned selected genera. This generated a null distribution for the number of genera selected in at least 6 of 10 runs under the same per-run selection burden.

The observed number of genera selected in at least 6 of 10 runs was compared with this null distribution. The ≥6/10 threshold was interpreted as a descriptive stability tier rather than a formal significance threshold. At the individual-genus level, empirical p values were computed by comparing each genus-specific selection frequency with its randomized null distribution.

## Supplementary Methods 12. Predictive evaluation

For the main predictive evaluation, the labeled target cohort was randomly split into a training set comprising 70% of samples and a held-out test set comprising the remaining 30%. Model discrimination was evaluated using AUC on the held-out test set. Each scenario was repeated across 10 random seeds to account for training variability.

The main BRIDGE-KO configuration used ComBat applied to both source and target data and the top 25% most proximal source samples for covariance estimation. A schematic workflow illustrates the complete BRIDGE-KO procedure, including source covariance estimation, knockoff generation, paired original-knockoff input construction, supervised DNN training, held-out target evaluation, and feature-level contrastive scoring.
